# Reactive Inkjet Printing of Regenerated Silk Fibroin Films for Use as Dental Barrier Membranes

**DOI:** 10.3390/mi9020046

**Published:** 2018-01-27

**Authors:** Patrick M. Rider, Ian. M. Brook, Patrick J. Smith, Cheryl A. Miller

**Affiliations:** 1 School of Clinical Dentistry, The University of Sheffield, Sheffield S10 2TA, UK; prider1@sheffield.ac.uk (P.M.R.); i.brook@sheffield.ac.uk (I.M.B.); c.a.miller@sheffield.ac.uk (C.A.M.); 2 Department of Mechanical Engineering, The University of Sheffield, Sheffield S1 3JD, UK

**Keywords:** regenerated silk fibroin, reactive inkjet printing, silk crystallinity, degradation rate, tissue engineering scaffolds, dental implantology, nano-hydroxyapatite

## Abstract

Current commercially available barrier membranes for oral surgery have yet to achieve a perfect design. Existing materials used are either non-resorbable and require a second surgery for their extraction, or alternatively are resorbable but suffer from poor structural integrity or degrade into acidic by-products. Silk has the potential to overcome these issues and has yet to be made into a commercially available dental barrier membrane. Reactive inkjet printing (RIJ) has recently been demonstrated to be a suitable method for assembling silk in its regenerated silk fibroin (RSF) form into different constructs. This paper will establish the properties of RSF solutions for RIJ and the suitability of RIJ for the construction of RSF barrier membranes. Printed RSF films were characterised by their crystallinity and surface properties, which were shown to be controllable via RIJ. RSF films degraded in either phosphate buffered saline or protease XIV solutions had degradation rates related to RSF crystallinity. RSF films were also printed with the inclusion of nano-hydroxyapatite (nHA). As reactive inkjet printing could control RSF crystallinity and hence its degradation rate, as well as offering the ability to incorporate bioactive nHA inclusions, reactive inkjet printing is deemed a suitable alternative method for RSF processing and the production of dental barrier membranes.

## 1. Introduction

Periodontitis is a dental disease which damages the supporting structures of teeth, such as the alveolar bone, and can lead to eventual tooth loss. Periodontitis has been shown to be present in a mild to severe form in 24.4% of adults aged between 30 and 34 years, which increases to 70.1% prevalence in adults aged 65 years and over [1]. If periodontitis is not treated early enough, or the periodontal condition continues to decline, it may be necessary for surgical intervention. Barrier membranes can be used in conjunction with guided bone regeneration (GBR) to help repair the damage caused by periodontitis. GBR promotes and directs the growth of new bone, whilst the barrier membrane secludes the defect site from infiltration by fast-growing connective and epithelial tissues which would otherwise fill the defect space. In the field of implantology, dental barrier membranes are used to aid with the fixation of dental implants in over 40% of implantations to improve bone augmentation [2].

The ideal properties for a barrier membrane are: to have a controllable degradation rate; to be biocompatible; to prevent surrounding tissues from collapsing into the defect space; and to provide cell occlusivity [2].

Current commercial barrier membranes are produced out of materials which are either non-resorbable and require a secondary surgery for their extraction, or made from resorbable materials which can have poor structural integrity or degrade into acidic by-products [3]. Silk could be considered as a possible alternative material as it already has a long history of use as a medical material [4,5]. More recently, research into silk for tissue engineering applications has increased due to the development of regenerated silk fibroin (RSF) structures, such as sponges, films, hydrogels, and mats [6,7]. Silk will only degrade in the presence of enzymes and degrades into non-harmful free amino acids and peptides [8,9]. Many structural characteristics, such as biodegradation rate and mechanical strength, can be adapted by using regenerated silk fibroin [10,11,12]. These adaptable properties make it ideal for use as a barrier membrane or tissue engineering scaffold material where control over all aspects of the material is required. Other properties of silk which make it a desirable material include versatility of sterilisation techniques [7,13,14], solvent- and water-based processing, and the ability to modify chemical groups along its structure [15].

Silk fibroin has several polymorphs: silk I, silk II, and silk III. Silk I has an unordered structure which is water soluble and present within the silkworm gland before spinning; silk II has a crystalline structure that is non-water soluble and produced during spinning from the silk worm spinneret; silk III is an unstable structure which forms at the water–air interface. As regards the current research, silk I and silk II are of interest.

Silk I consists of an unordered fibroin structure, mostly composed of α-helix and random coils, whilst silk II is mostly composed of a crystalline β-sheet structure. Silk I can be transformed into silk II by exposure to methanol or potassium chloride [16,17,18], stretching [19], as well as through heat treatments [20]. Methanol dehydrates the unordered random coil structural component of silk I, converting it into anti-parallel β-sheets and thereby creating a water-insoluble silk II structure [16,17]. The ability to change silk I to silk II make it ideal for processing and manufacture.

Current methods for producing RSF films involve either casting, spin drying, or electrospinning; however, these methods have a limited control over the final structure and require additional procedural steps to treat the films and improve their mechanical properties [6]. Reactive inkjet printing offers complete control over film design and structure, as well as the possibility of combining film manufacture with a methanol treatment to induce β-sheet crystallinity. Previously, we have reported the reactive inkjet printing of RSF, where we have demonstrated the flexibility in film design and production when using an inkjet printer [21]. This paper will establish the characteristics of RSF solutions for inkjet printing and printed RSF films for use in tissue engineering, and more specifically for dental barrier membranes. We have also produced films with the inclusion of nano-hydroxyapatite (nHA). The inclusion of nHA within a tissue engineering construct has been shown to improve osteogenic activity and therefore improve bone cell interaction [22,23]. The ability to include bioactive components, such as nHA, could prove beneficial for improving site regeneration after periodontal surgery.

## 2. Materials and Methods

### 2.1. Silk Fibroin Extraction

Regenerated silk fibroin (RSF) was extracted from Bombyx Mori silkworm cocoons (Wild Fibres, Birmingham, UK) based on the protocol described by Rockwood et al. [24]. Briefly, silkworm cocoons were cut open and the silkworm extracted. Silk fibres were released from the cocoons by boiling them in a 0.02 M sodium carbonate (≥99.5% purity, ACS reagent, Sigma Aldrich, Dorset, UK) alkaline solution for 30 min. The fibres were thoroughly rinsed with distilled water three times before being dried out. The silk fibres were added to a 9.3 M lithium bromide (LiBr) (≥99% purity, ReagentPlus^®^, Sigma Aldrich, UK) solution and heated in an oven at 70 °C for 3 h 30 min, as recommended by Sah et al. [25], by which time the silk fibres had completely dissolved to form an RSF solution. To remove the LiBr from the RSF solution, the solution was dialysed against 1 L of distilled water using a 3–12 mL dialysis cassette (Slide-A-Lyzer™ Dialysis Cassettes, 3.5 K molecular weight cut-off (MWCO), 12 mL, ThermoFisher Scientific, Loughborough, UK) over a period of 72 h with frequent water changes. To remove any contaminants still present within the RSF solution, such as remaining silkworm, the solution was centrifuged at 13,000 G for 20 min at 4 °C. To concentrate the solution, it was dialysed against a 5 wt % poly (ethylene glycol) (Av. mol. wt 10,000, Sigma Aldrich, UK) using a 0.5–3 mL dialysis cassette (Slide-A-Lyzer^™^ Dialysis Cassettes, 3.5 K MWCO, 3 mL, ThermoFisher Scientific, Loughborough, UK) over 20 h. The concentrated RSF solution was then diluted down using distilled water to desired concentrations and stored at 4 °C.

### 2.2. Nano-Hydroxyapatite Synthesis

Nano-hydroxyapatite (nHA) was synthesised using a wet precipitation method [26] based upon the patented Fluidinova process [27]. A 4.0827 g amount of potassium phosphate monobasic (≥99.0%, powder, Sigma Aldrich, UK) was dissolved in 250 mL distilled water, and 7.3506 g of calcium chloride (United States Pharmacopeia (USP) testing specification, Sigma Aldrich, UK) was dissolved in 500 mL distilled water in a separate beaker. A 1 M potassium hydroxide (American Chemical Society grade (ACS) reagent, ≥85%, pellets, Sigma Aldrich, UK) solution was gradually added to both solutions to increase their pH. The potassium phosphate solution had its pH increased up to a pH of 13, whilst the calcium chloride solution had potassium hydroxide solution added to it until the solution became continuously cloudy, which occurred at a pH of 12.8. The two solutions were combined in a fast and fluid motion, and were mixed with the magnetic stirrer for 1 h. After a 7 h rest period, the solution had started to separate into a cloudy solution which sank to the bottom of the beaker while a clear solution remained at the top. The clear solution was syphoned off, the beaker was topped up with distilled water, and the solution was mixed again with a magnetic stirrer for 20 min. The solution was again left to rest and the rinsing process repeated twice to neutralise the solution. After the final wash, the denser cloudy solution at the bottom of the beaker was then used for printing. nHA content was confirmed with X-Ray Diffraction and Fourier Transform Infrared Spectroscopy.

To make the nHA/RSF inks, the nHA slurry was mixed with RSF solution which had a concentration of 100 mg·mL^−1^. nHA/RSF ink concentrations were calculated based upon the dry weight concentration of nHA for each solution. Inks were made which had dried weights equivalent to 100%, 75%, 50%, and 25% nHA content. From this point on, each of the nHA/RSF inks will be referred to based upon their dried weight nHA content.

### 2.3. Ink Analysis

Ink viscosities and surface tensions were measured to calculate their respective Z numbers. Viscosity measurements were made using a rheometer (Physica MCR 301 Rheometer, Anton Paar, St Albans, UK) with a cone and plate geometry (θ = 0.998°; diameter 49.972 mm; gap set to 0.1 mm) at a temperature of 20 °C. Viscosity measurements were made in the rotational mode between 0.01 and 10,000 s^−1^.

Surface tension measurements were made using the Pendant Drop method. Droplets were filmed as they detached from a vertically positioned flat-tipped needle. Images of the droplet just before detachment were analysed using a Pendant Drop plugin [28] on open-sourced Fiji software [29].

Z numbers were calculated using the inverse of the Ohnesorge number (Oh), shown in Equations (1) and (2).

(1) $$Oh=\;\frac{\eta }{\sqrt{\left(\rho \gamma a\right)}}$$

(2) $$Z\;number=\;\frac{1}{Oh}$$

where ‘𝜂’ is the viscosity of the ink, ‘𝜌’ is the density of the fluid, ‘𝛾’ is the surface tension, and ‘a’ is the radius of the nozzle. Viscosity measurements used to calculate Z numbers were taken from the infinite viscosity range for each of the inks.

### 2.4. Inkjet Printing

Inkjet printing was performed on a MicroFab drop-on-demand piezoelectric inkjet printer using JetLab4 software. A piezo printhead with a nozzle aperture of 80 µm was used to print the RSF and nHA/RSF inks, and a 60 µm printhead was used to print methanol (ACS reagent, ≥98%, Sigma Aldrich, UK). Jetting parameters were optimised for each ink to get a stable droplet formation. Printing was performed at room temperature, which remained close to 20 °C. Samples were printed “on the fly”, meaning that the substrate was in constant motion below the printhead. Printing parameters varied slightly between RSF batches and as the solution aged. After two weeks stored at 4 °C, the RSF ink became unreliable and was likely to block the nozzle. Therefore, no inks were used which were over a fortnight old.

**Figure 1:**
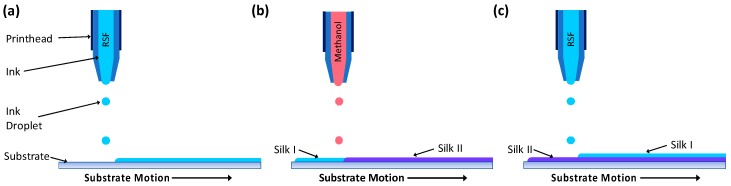
Schematic showing the printing of layers to produce a regenerated silk fibroin (RSF) film; (**a**) a layer of RSF is printed, (**b**) followed by a layer of methanol which converts the RSF structure from silk I to silk II, (**c**) the process is repeated, with a layer of RSF printed on top of the previous layer.

RSF films were produced by printing RSF ink at a concentration of 100 mg·mL^−1^ with a droplet step size between 0.14 and 0.16 mm and methanol with a step size between 0.06 and 0.17 mm onto 13 mm diameter glass coverslips (Circular coverglasses, Agar Scientific, Stansted, UK). To produce films with different crystallinities, RSF films were printed with different volumes of methanol. Methanol volume was controlled by printed droplet density, whereby the distance between methanol droplets was varied to change the volume of ink printed per unit area. Droplet volumes were calculated using photographs of the ejected droplets and the equation for the volume of a sphere. Each printed RSF layer was followed by a subsequent layer of printed methanol as demonstrated in Figure 1. Films were produced by printing alternate layers of RSF ink and methanol to a height of 20 RSF layers.

### 2.5. Film Characterisation

RSF films were analysed for their crystallinity by initially measuring their secondary protein structure using Fourier transform infrared spectroscopy with attenuated total reflection (FTIR-ATR) (Frontier FTIR, PerkinElmer, Seer Green, UK equipped with a Golden Gate™ Diamond ATR, Specac, Orpington, UK). Each measurement was comprised of 16 accumulated scans between wavenumbers of 4000–600 cm^−1^ with a resolution of 4 cm^−1^. Fourier self-deconvolution (FSD) performed on the spectra to calculate RSF crystallinity. FSD calculates the contribution of the secondary protein structures to the overall RSF structure. FSD was performed on the amide I spectral region (1705–1595 cm^−1^) using OriginPro 2016 software, using the methodology explained by Hu et al. [20] and the β-sheet structure used as RSF crystallinity.

The contact angle of water droplets on the RSF films was measured from photographs (camera composed of a Macro 10X lens, Computar, Cary, NC, USA and a DCC1545M camera, Thorlabs, Newton, NJ, USA) using ImageJ2 software [30] with the plugin Dropsnake [31]. Contact angle measurements were given for both sides of the droplet, and an average of both measurements was recorded. Five repeats were performed for each droplet. The roughness of the RSF films was measured using an interferometer (ContourGT, Bruker, Coventry, UK). Surface area roughness (S_a_) was calculated over a 0.6 mm × 0.47 mm area from three different points on the RSF films.

Degradation tests were performed in either phosphate buffered saline (PBS) (Dulbecco’s Phosphate Buffered Saline, without calcium chloride and magnesium chloride, Sigma Aldrich, UK) or a protease (Protease XIV, 3.5 units/mg, from Streptomyces griseus, powder, Sigma Aldrich, UK) solution. The solution comprised of protease XIV in PBS at a 0.1 mg·mL^−1^ concentration as reported by Pritchard et al. [32]. RSF films were placed into 24-well plates and submerged in either 1 mL of PBS or 1 mL protease solution and incubated at 37 °C for a maximum period of 8 days, with solutions replaced daily. At designated time points, 1, 2, 3, 5, and 8 days, a subset (*n* = 3) of the films were removed. The removed films were washed three times by submersion in 1 mL of PBS for 2 min. Films were then dried in a drying oven at 60 °C for 1 h to remove moisture. Films were weighed using an analytical balance prior to and after the degradation test.

## 3. Results

### 3.1. Ink Characterisation

**Figure 2:**
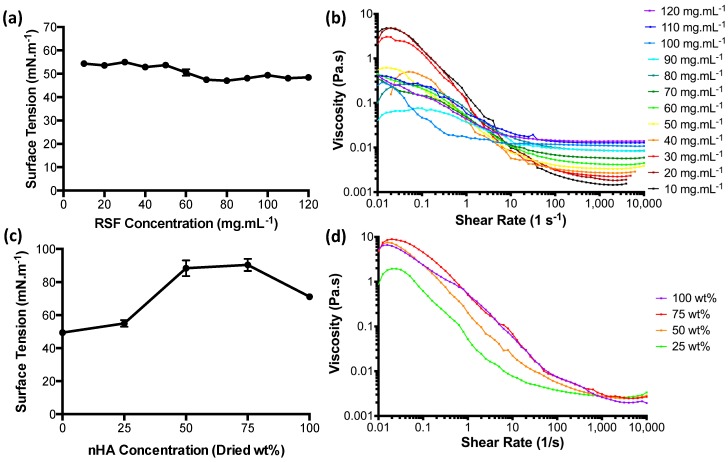
(**a**) Surface tension measurements for RSF inks, (**b**) apparent viscosity of RSF inks, (**c**) surface tension measurements of nHA/RSF inks, and (**d**) apparent viscosity of nHA/RSF inks.

The surface tension values for the RSF inks at room temperature (Figure 2a) were found to lie between 47 and 55 mN·m^−1^. Surface tensions appear to change according to concentration. Low concentration RSF inks of 50 mg·mL^−1^ and below had the highest surface tensions with an average of 53.5 mN·m^−1^. RSF concentrations of 70 mg·mL^−1^ and higher had an average surface tension of 48.1 mN·m^−1^.

The apparent viscosities for the RSF inks are shown in Figure 2b. The RSF solutions appear to be unstable at shear rates below 10 s^−1^ and experienced a non-Newtonian behaviour with pronounced shear thinning up to shear rates of 1000 s^−1^. From a shear rate of 100 s^−1^ and above, the solutions began to become shear-independent, transitioning into Newtonian behaviour. A comparison of the infinite viscosities (the region of high shear where viscosity has become shear-independent) shows a linear increase with increased concentration.

Surface tension measurements for the nHA/RSF inks showed significant differences between each concentration in Figure 2c. nHA/RSF ink 100% (pure nHA without RSF content) had a surface tension of 71 mN·m^−1^, similar to pure water, which is 72 mN·m^−1^ at room temperature [33]. nHA/RSF ink 25% had a higher surface tension than pure RSF at a concentration of 100 mg·mL^−1^ (shown as 0% on the graph) by about 5 mN·m^−1^. nHA/RSF inks 50% and 75% had significantly higher surface tensions than the other nHA/RSF inks, with average surface tensions of 88 and 90 mN·m^−1^, respectively.

The apparent viscosities of the nHA/RSF inks, shown in Figure 2d, appear to be more stable at low shear rates in comparison to the pure RSF inks. Shear independence for all the inks had been achieved by a shear rate of 2000 s^−1^.

**Figure 3:**
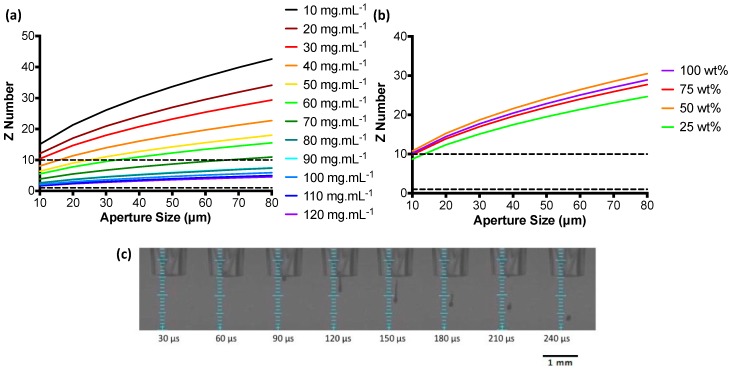
Z numbers over a range of nozzle diameters for (**a**) RSF inks and (**b**) nHA/RSF inks, the horizontal dashed line represents the range of Z numbers with predicted stable printing; (**c**) Droplet formation of RSF ink at a concentration of 100 mg·mL^−1^ taken at 30 μs intervals.

Z numbers were used to characterise the RSF inks over a range of concentrations and give an indication as to their printability. Low Z numbers indicate a viscous ink, whilst an ink with a high Z number is prone to produce satellite droplets [34]. Z numbers over a range of nozzle aperture sizes are shown in Figure 3a,b. Z number values are seen to increase as the nozzle aperture diameters become larger. RSF inks with the highest concentrations fall within the region of reliable droplet formation as defined by the Z number. For the largest aperture size (80 μm), RSF inks 90, 100, 110, and 120 mg·mL^−1^ had a Z number between 1 and 10. Z numbers for the nHA/RSF inks show that none of the inks fell within the predicted “most stable droplet” range above an aperture size of 10 μm.

All RSF inks with a Z number between 1 and 10 for an aperture size of 80 μm were test printed. An 80 μm printhead was chosen to print the inks, as it would produce the largest droplets and therefore be the fastest at depositing large quantities of material. Stable droplet formation, depicted in Figure 3c, was achieved with all concentrations tested. However, RSF inks 110 and 120 mg·mL^−1^ crusted over after long periods of printing and were therefore discarded and RSF ink 100 mg·mL^−1^ was chosen to print the RSF films.

Although none of the nHA/RSF inks gave a Z number between 1 and 10 for aperture sizes above 10 μm, they were all test printed with an 80 μm diameter printhead. Each ink produced a stable droplet formation without the formation of satellite droplets. The Z number can be used for guidance when evaluating the printability of inks, as previously Tekin et al. [35] have reported the printing of solvents with Z numbers as high as 91. Therefore, all nHA/RSF inks were used to produce the nHA/RSF films.

The conversion of silk I to silk II required printing methanol. Z numbers were calculated for methanol to estimate droplet stability using surface tension and viscosity values at 25 °C taken from the work of Won et al. [36]. Z numbers calculated using these values were higher than the most stable Z number range. To test for droplet stability, methanol was loaded into the printer and jetted through a nozzle with an 80 μm diameter. Droplet formation was found to be unstable with the formation of satellite droplets. To improve droplet stability, methanol was printed through a 60 μm diameter printhead, which had a lower Z number. A stable droplet could be produced using the smaller printhead and was therefore selected to print the methanol for the RSF and nHA/RSF films.

### 3.2. RSF Film Characterisation

**Figure 4:**
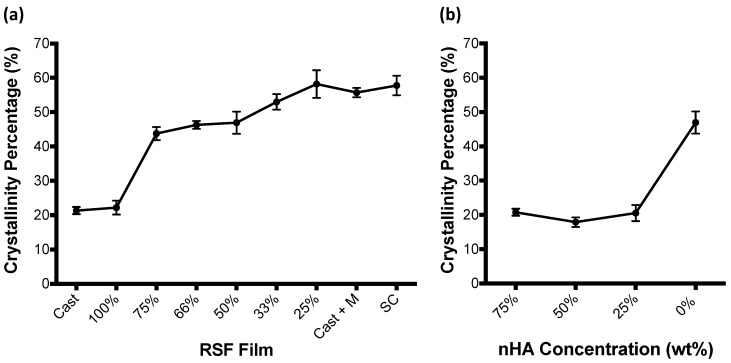
RSF crystallinity for (**a**) pure RSF films and (**b**) nHA/RSF films. nHA/RSF film 0% represents RSF film 50% and is used as a comparison. Crystallinity data for pure RSF films previously published [21]. Cast + M: cast film which has been submerged in methanol for 4 days; SC: unprocessed Bombyx Mori silkworm cocoon.

RSF film crystallinity, calculated by Fourier deconvolution of the amide I region of FTIR-ATR spectra, is shown to increase with increasing methanol volumes in Figure 4a. Percentages shown in the graph represent the contributing volume of RSF ink used to produce the film, whereby RSF film 100% is a film which has been entirely produced out of the RSF ink without the inclusion of methanol, and RSF film 25% represents a film which has been produced with a 3:1 volume ratio of RSF ink to methanol, and has therefore had the largest volume of methanol printed onto its surface. Cast + M represents a cast film which has been submerged in methanol for 4 days. SC is the unprocessed Bombyx Mori silkworm cocoon. Both RSF films without methanol treatment (Cast and 100%) have similar levels of crystallinity. Between RSF films 100% and 75%, film crystallinities increase from ~20% to ~44%. The degree of film crystallinity remains similar between RSF films 75% and 50%, before steadily increasing between RSF films 50% and 25%. Crystallinity of the RSF films increased by 6% between RSF film 50% and RSF film 33%, and then by a further 5% between RSF film 33% and RSF film 25%. RSF film 25%, which had been made with the largest volume of methanol, had a similar crystallinity to that of a cast RSF film submerged in methanol for 4 days and that of an unprocessed Bombyx Mori silkworm cocoon.

RSF crystallinity was also compared between the nHA/RSF films and are shown in Figure 4b. Crystallinities were calculated using Fourier deconvolution of the FTIR-ATR spectra amide I region (1705–1595 cm^−1^). This region of the FTIR-ATR spectrum was not affected by the presence of the nHA particles. Films which included the nHA particles had significantly lower crystallinities than that of the pure RSF film (nHA/RSF film 0%), which had a 50% ratio of RSF solution to methanol. Instead, RSF crystallinity is similar to that of pure RSF films without exposure to methanol, as seen in Figure 4a (RSF films Cast and 100%).

**Figure 5:**
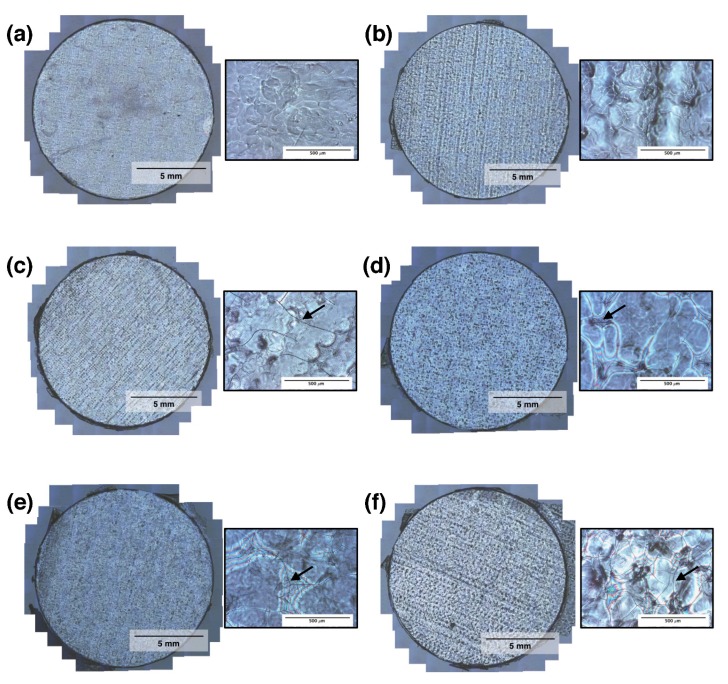
Light microscopy photographs of the RSF films: (**a**) 100%, (**b**) 75%, (**c**) 66%, (**d**) 50%, (e) 33%, and (**f**) 25%. Next to each film is a photo of the film at a higher magnification. Arrows are used to highlight cracks in the higher magnification photos.

The light microscopy photographs of the RSF films in Figure 5 show that each film had a highly textured surface. There are clear troughs and peaks visible in RSF films 75% and 66% (Figure 5b,c), which have been produced by the deposition of droplets along the direction of printing. Lines of droplets are less visible in RSF films 50%, 33%, and 25% (Figure 5d–f, respectively), although peaks and troughs are still seen as light and dark patches. Under higher magnification, nano-scale cracks are visible which increase in size and density with increasing volumes of printed methanol.

**Figure 6:**
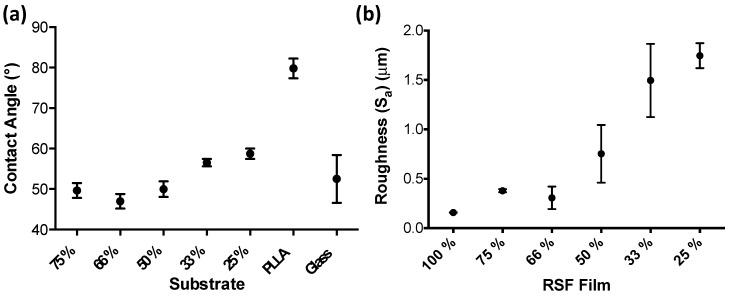
(**a**) Contact angle measurements for the RSF films and that of controls PLLA and glass, (**b**) Surface roughness S_a_ of the RSF samples measured using interferometry, *n* = 5.

Water droplet contact angles were measured on the RSF films as well as on comparisons of poly(L-lactide) (PLLA) and glass coverslips, and are shown in Figure 6a. The average contact angle for each of the RSF films became larger with increasing crystallinities, except between RSF films 75% and 66%. The average contact angles were 49.7°, 47°, 50°, 56.6°, and 58.7° for RSF films 75%, 66%, 50%, 33%, and 25%, respectively.

RSF films had their surface roughness measured using interferometry. Surface area roughness measurements (S_a_) show that RSF films 100%, 75%, and 66% have similar levels of roughness, below 0.5 um. However, increasing concentrations of printed methanol induces rougher surfaces, which becomes more pronounced with the more crystalline films. Surface roughness reaches a peak value of 1.75 um with RSF film 25%.

**Figure 7:**
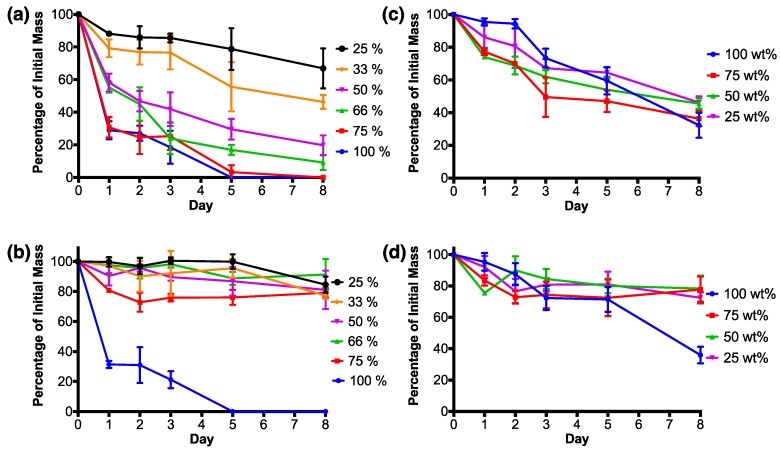
Degradation graphs for the RSF films (**a, b**) and nHA/RSF films (**c, d**), degraded in a solution of Protease XIV (**a, c**) or phosphate buffered saline (PBS) (**b, d**). Percentages show the mass of each film in proportion to its original mass, along with the standard deviation for each value. RSF film degradation data previously published [37].

RSF films degraded with protease XIV experienced their largest mass loss by the first day (Figure 7a). RSF films 100% and 75% both lost around 30% of their initial mass, whilst RSF films 66% and 50% lost around 55–58% of their initial masses. The most crystalline films, RSF films 33% and 25%, experienced the smallest mass losses by the first day in comparison to the other RSF films. After the first day, degradation rates reduced and kept at a relatively steady rate.

By day 5, RSF film 100% had completely degraded, which was followed by RSF film 75% on day 8. By the final day, the remaining films had masses relative to their crystallinity, whereby higher crystallinity films had the lowest percentage mass loss when degraded in protease XIV. Films with the smallest to highest remaining masses were as follows, RSF films 66% < 50% < 33% < 25%, each losing around 90%, 80%, 55%, and 35%, respectively, of their initial mass.

RSF films 100% and 75% were the only RSF films to experience a large mass loss by the first day when degraded in PBS in comparison to the other RSF films (Figure 7b). RSF film 100% had a similar degradation rate when degraded with either protease XIV or PBS and was completely degraded after 5 days. RSF films degraded in PBS had fluctuating masses over the 8 day degradation test, and by the final day, had lost a maximum of around 20% of their initial masses. Final masses were not related to film crystallinities.

Figure 7c shows that nHA/RSF films which consisted purely of printed nHA (nHA/RSF film 100%), when degraded with protease XIV, experienced a low amount of mass loss over the first 2 days of the degradation study, after which significantly large mass drops occurred between each time point. Degradation of the remaining nHA/RSF films degraded with protease XIV experienced two periods with a large mass loss. The first occurred by the first day, and the second between days 2 and 3. Of the nHA/RSF films which included RSF within their structure, nHA/RSF film 75% lost the most mass by day 8, whilst nHA/RSF films 50% and 25% finished with similar mass losses. nHA/RSF films with RSF content (nHA/RSF films 75%, 50%, and 25%) finished with similar mass losses after 8 days of degradation in PBS solution (Figure 7d). The relative masses of these films fluctuated over the course of the 8 day period, possibly caused by different amounts of nHA being released into solution. nHA/RSF film 100% experienced similar degradation profiles in both the PBS and protease XIV solutions.

## 4. Discussion

Droplet formation and stability are key to producing reliable and repeatable experiments with reactive inkjet printing. The two key factors which influence droplet formation and stability are the applied waveform and the rheology of the ink. It was therefore important to analyse the RSF and nHA/RSF inks before using them for printing. Viscosity has been linked to the stability of droplets by preventing instabilities from forming before droplet detachment [38,39,40]; however, viscosities which are too high will dampen out acoustic waves before a droplet is formed. Surface tensions are required to hold a meniscus at the nozzle and prevent flooding of the nozzle tip. High surface tensions will cause faster separation of the droplet from the nozzle as well as larger droplet formation [38].

RSF inks showed a slight drop in surface tensions with increased concentration. This could be explained by the work of Yang et al. [41], who modelled the RSF protein at the liquid-air interface and suggested two separate models for high and low RSF concentrations. RSF molecules were modelled as multi-block amphiphilic macromolecules, which at low concentrations are arranged into helical silk III or β-sheet silk II conformations at the liquid-air interface, and produce a high surface elasticity. As the concentration of the RSF solutions increases, the air-water interface becomes more crowded with RSF molecules. A lack of space at the surface causes RSF molecules to protrude out of the surface and into a hairpin-like configuration, decreasing surface elasticity [41]. The experiment by Yang et al. looked at RSF concentrations over a much larger range than those used in this study; however, it is suggested that the slight drop in surface tension experienced by the higher concentrated solutions could be the result of changing concentrations of RSF molecules with a hairpin-like conformation at the liquid-air interface.

Surface tensions of the nHA/RSF inks showed significant changes with each nHA concentration. The ink containing only nHA and no RSF (nHA/RSF ink 100%) had a slightly higher surface tension than water, and the addition of 25 dried wt % nHA to RSF only caused a slight increase of surface tension. However, the combination of both nHA and RSF, where the dried nHA weight concentration was 50% and 75%, created a synergistic effect which caused a significant increase in surface tension.

Over the range of dynamic viscosity measurements, the RSF inks appeared to be less stable at low concentrations. However, as the shear rate increased and the inks approached shear independence, the inks became more stable. At shear rates around 100 s^−1^, the RSF inks began to form a Newtonian plateau and by a shear rate of 1000 s^−1^ had become shear-independent. The nHA/RSF inks were more stable at low concentrations in comparison to the RSF inks; however, they took longer to reach shear independence. Both RSF and nHA/RSF inks had reached shear independence by a shear rate of 2000 s^−1^, which is a mid-range shear rate experienced during printing [42]. As a shear rate of 2000 s^−1^ is comparable to inkjet printing forces, viscosity measurements for Z number calculations were taken from this position.

When Newtonian fluids are analysed for printing, the zero viscosity (the viscosity at a very low shear rate) is used to calculate the Z number. However, for non-Newtonian fluids the viscosity becomes a function of shear, whereby the zero viscosity can be vastly different to that of the infinite viscosity. According to Yoo et al., the ejection of a droplet is associated with the infinite shear viscosity and not the zero viscosity [43]. Therefore, all Z number calculations used the infinite viscosity value and the instability of the RSF inks at low concentrations was not considered a problem.

Z numbers correctly predicted a stable droplet formation for the RSF inks. However, the highest concentration inks were susceptible to crusting-over during printing and therefore RSF ink 100 mg·mL^−1^ was chosen as the ideal ink to produce the RSF films. The Z numbers for the nHA/RSF inks with an aperture size of 80 μm were all well above the predicted stable range. However, all inks were tested for printing with an 80 μm printhead and each ink was shown to have a stable droplet formation. Therefore, all nHA/RSF inks were considered suitable for further printing.

The crystallinity data showed that a small volume of methanol could induce a large proportion of the RSF to become crystalline. The degree of RSF crystallinity doubled between RSF films without any methanol treatment (RSF films Cast and 100%) and the RSF film which had been exposed to the smallest volume of methanol (RSF film 25%). However, after this initial transition, RSF crystallinity did not significantly change up until RSF film 50%, which was produced with a 1:1 volume ratio of RSF to methanol. Significant changes in crystallinity were then observed between RSF films 50% and 25%. A peak crystallinity was reached with RSF film 25% which was similar to a cast RSF film, submerged in methanol for 4 days, as well as that of a native Bombyx Mori silkworm cocoon, suggesting a complete transition of silk I to silk II. The crystallinity data also showed that printing alone without methanol treatment did not induce crystallinity due to shear, as there was no difference in crystallinity between RSF films Cast and 100%. This is important, as it demonstrates that all structural changes observed by the printing of different volumes of methanol are caused by interactions with the methanol alone.

The inclusion of nHA within the composite ink was shown to affect the transition of silk I to silk II, as nHA/RSF films showed similar RSF crystallinity to that of RSF films without methanol treatment. Additional research by the authors showed that Fourier deconvolution of the nHA/RSF films had a larger volume of β-turns compared to the pure RSF films [44]. Previously, Yamane et al. suggested that β-turns are a precursor to a β-sheet structure [45]. This is supported by Wilson et al. who created a model amorphous fibroin peptide chain, which, when exposed to methanol, gradually transitioned into a crystalline silk II structure. During the transition, an intermediate state appeared which consisted of a high proportion of β-turns [46]. This could suggest that the presence of nHA within the composite ink was hindering the conversion of silk I to silk II, possibly by reduced contact with the methanol, and that only a partial transition occurred.

The peaks and troughs on the RSF films visible in the light microscopy photos were caused by the spacing of the printed RSF droplets. The initial spacing of the droplets was chosen to produce a uniform layer and create a flat film. As multiple layers of RSF were printed, the RSF droplets were no longer interacting with the glass coverslip and were instead interacting with dried RSF film. As lines of droplets are visible on the surface of the films, it would indicate that the RSF films were slightly more hydrophobic than that of the glass coverslips, causing the droplets to spread over a smaller area. The only film where no droplets were visible was on RSF film 100%, which had had no methanol treatment. No visible droplets on the surface of RSF film 100% could be an indication that the methanol treatment was causing the films to become more hydrophobic, which makes sense when one considers that the addition of methanol results in the production of insoluble silk II.

Significant cracking of the films, which began to form on RSF film 66%, became larger and more frequent up until RSF film 25%. As there are no cracks visible on RSF film 100%, the cracking could be the result of rapid dehydration of the RSF caused by methanol. Larger volumes of methanol would have had longer to diffuse into the RSF before evaporating and therefore caused more prolific crack propagation. The effects of crack formation on the surface of the films will have to be monitored for further development of RSF barrier membranes. A rougher surface could aid with cellular interactions; however, the cracking may cause problems with the structural integrity of the membranes.

Crystallinity of the RSF films was shown to influence water droplet contact angles. When RSF is in an amorphous state, polar groups along the molecule have a random orientation which produces a high surface energy and a more hydrophilic surface. During crystallisation, the polar groups are used for hydrogen bonding to produce a β-sheet structure [47]. As the polar groups are positioned within the β-sheet layers, the surface energy is reduced which increases hydrophobicity.

As observed with the light microscopy photographs, films which included larger volumes of methanol had the roughest surfaces. Increasing the volume of methanol caused larger, deeper, and more frequent cracking to occur and could be observed visually in Figure 5, which caused the roughness values of the RSF films to increase. The cracking of the films was most likely caused by the rapid dehydration of the RSF and was proportional to the volume of methanol printed.

Degradation of the RSF films was studied and compared by immersing them in either an enzymatic solution of protease XIV or in phosphate buffered saline (PBS) over an 8 day period. The enzymatic solution facilitated the breakdown of the fibroin structure and therefore produced faster degradation rates. Degradation within the PBS solutions should show the proportion of RSF films being actively broken down by enzymatic activity and how much RSF is lost simply due to dissolution of the water-soluble structures.

The largest mass loss for RSF films degraded with protease XIV had occurred by day 1, and was proportional to film crystallinity. Crystallinity continued to affect the degradation of the films, as by the final day of the protease XIV degradation study, mass loss was shown to be related to film crystallinity. It was expected that the RSF films degraded in PBS would have experienced mass losses by the first day which related to film crystallinity, as the non-crystalline water-soluble silk structures were dissolved. However, it was only RSF film 100%, which had had no methanol treatment, which experienced similar degradation rates in both degradation media.

A potential reason for the methanol-treated RSF films having different degradation profiles could be due to the way the films are produced. During printing, each printed layer of RSF solution is very thin, and it is necessary to print multiple layers to build up the mass of the film. A layer-by-layer approach to producing the films meant that layers of methanol were printed between sequential layers of RSF solution. Printing methanol between layers of RSF could have produced a film with a non-uniform structure, whereby layers of unordered silk I were encapsulated under layers of ordered silk II. Films exposed to larger volumes of methanol had higher crystallinities, which could represent thicker layers of silk II. Larger volumes of methanol would require longer to evaporate off the substrate. The longer evaporation times would increase RSF exposure to methanol, enabling it to diffuse further into the RSF film, converting unordered silk I into silk II. Therefore, films which have had a longer exposure to methanol would have thicker layers of silk II with denser crystal packing, encapsulating the unordered silk I beneath.

Degradation of the nHA/RSF films with protease XIV showed mass losses relative to the nHA concentration. Films with higher nHA contents were shown to experience larger mass losses. nHA would have been unsusceptible to proteolytic degradation; however, as the RSF degraded, it could have released the nHA into the surrounding solution. Therefore, the more concentrated films would have released larger quantities of nHA into the surrounding solution, which resulted in larger mass losses. The nHA/RSF films had also been shown to have a lower RSF crystallinity than that of the pure RSF films. Consequently, some of the mass loss could also be attributed to the dissolution of silk I content. The RSF did not degrade at the same rate as that of the pure RSF films with a similar crystallinity. This could be because of a higher β-turns content. β-turns are associated with a water-soluble silk I structure, however it has previously been shown that RSF films with a high β-turn content are water-insoluble [48]. nHA content was not shown to cause substantial differences between the degradation rates of the nHA/RSF films in PBS solution, except for the pure nHA film.

nHA/RSF film 100% experienced the largest mass loss in both the PBS and protease XIV solutions. This could have been caused by a lack of RSF binding the nHA crystals together, which, upon washing of the films, would be more vulnerable to becoming dislodged and washed away. The lack of structural stability of the printed nHA could explain the similarity of degradation rates of nHA/RSF film 100% in both degradation solutions.

## 5. Conclusions

Silk has long been used as a suture material, but it is only now, with the ability to process silk into different three-dimensional (3D) structures using a reconstituted silk fibroin solution (RSF), can it be used for a wider variety of medical applications. Its excellent biocompatibility, mechanical properties, controllable degradation rate, and non-toxic degradation by-products make it an attractive material for use as a dental barrier membrane. Current barrier membrane materials do not possess all the advantageous characteristics of RSF, making RSF an appealing choice for future designs.

In this paper, we have demonstrated that RSF and nHA/RSF solutions can be successfully printed using an inkjet printer. We have also shown that reactive inkjet printing can be used to control the structural characteristics of RSF, and gradually induce crystallinity. This is important in demonstrating that reactive inkjet printing may offer greater control over barrier membrane characteristics than that of other current RSF processing methods. For the first time, it has been shown how the reactive inkjet printing of RSF can be used to control degradation rates via film crystallinities. This major finding demonstrates the potential for reactive inkjet printing for producing RSF tissue engineering constructs. Control over RSF degradation rate could be beneficial in the development of an ideal barrier membrane, as RSF degradation rates could be matched to the rate of healing and site regeneration. The ability to incorporate bioactive components, for example nHA, within the printed films offers further potential of inkjet-printed films to aid with site recovery and the healing process. These properties make it ideal for producing future tissue engineering constructs, such as barrier membranes. Further work investigating the reactive-inkjet-printed barrier membranes and their interactions with soft and hard tissue cells is needed to be conducted to further demonstrate their suitability for tissue engineering scaffolds.
